# Recent discoveries in microbiota dysbiosis, cholangiocytic factors, and models for studying the pathogenesis of primary sclerosing cholangitis

**DOI:** 10.1515/med-2022-0481

**Published:** 2022-05-13

**Authors:** Yu Huang, Shuai Zhang, Jie-Feng Weng, Di Huang, Wei-Li Gu

**Affiliations:** Department of Surgery, Guangzhou First People’s Hospital, No. 1 Panfu Road, Yuexiu District, Guangzhou, Guangdong 510180, People’s Republic of China; Department of Surgery, Guangzhou First People’s Hospital, Guangdong 510180, People’s Republic of China

**Keywords:** dysbiosis, fibrosis, phenotype, inflammation, cholangiopathy

## Abstract

Primary sclerosing cholangitis (PSC) is a cholangiopathy caused by genetic and microenvironmental changes, such as bile homeostasis disorders and microbiota dysbiosis. Therapeutic options are limited, and proven surveillance strategies are currently lacking. Clinically, PSC presents as alternating strictures and dilatations of biliary ducts, resulting in the typical “beaded” appearance seen on cholangiography. The pathogenesis of PSC is still unclear, but cholangiocytes play an essential role in disease development, wherein a reactive phenotype is caused by the secretion of neuroendocrine factors. The liver–gut axis is implicated in the pathogenesis of PSC owing to the dysbiosis of microbiota, but the underlying mechanism is still poorly understood. Alterations in cholangiocyte responses and related signalling pathways during PSC progression were elucidated by recent research, providing novel therapeutic targets. In this review, we summarise the currently known underlying mechanisms of PSC pathogenesis caused by the dysbiosis of microbiota and newly reported information regarding cholangiocytes in PSC. We also summarise recently reported *in vitro* and *in vivo* models for studying the pathogenesis of PSC.

## 1 Introduction

**Figure 1:**
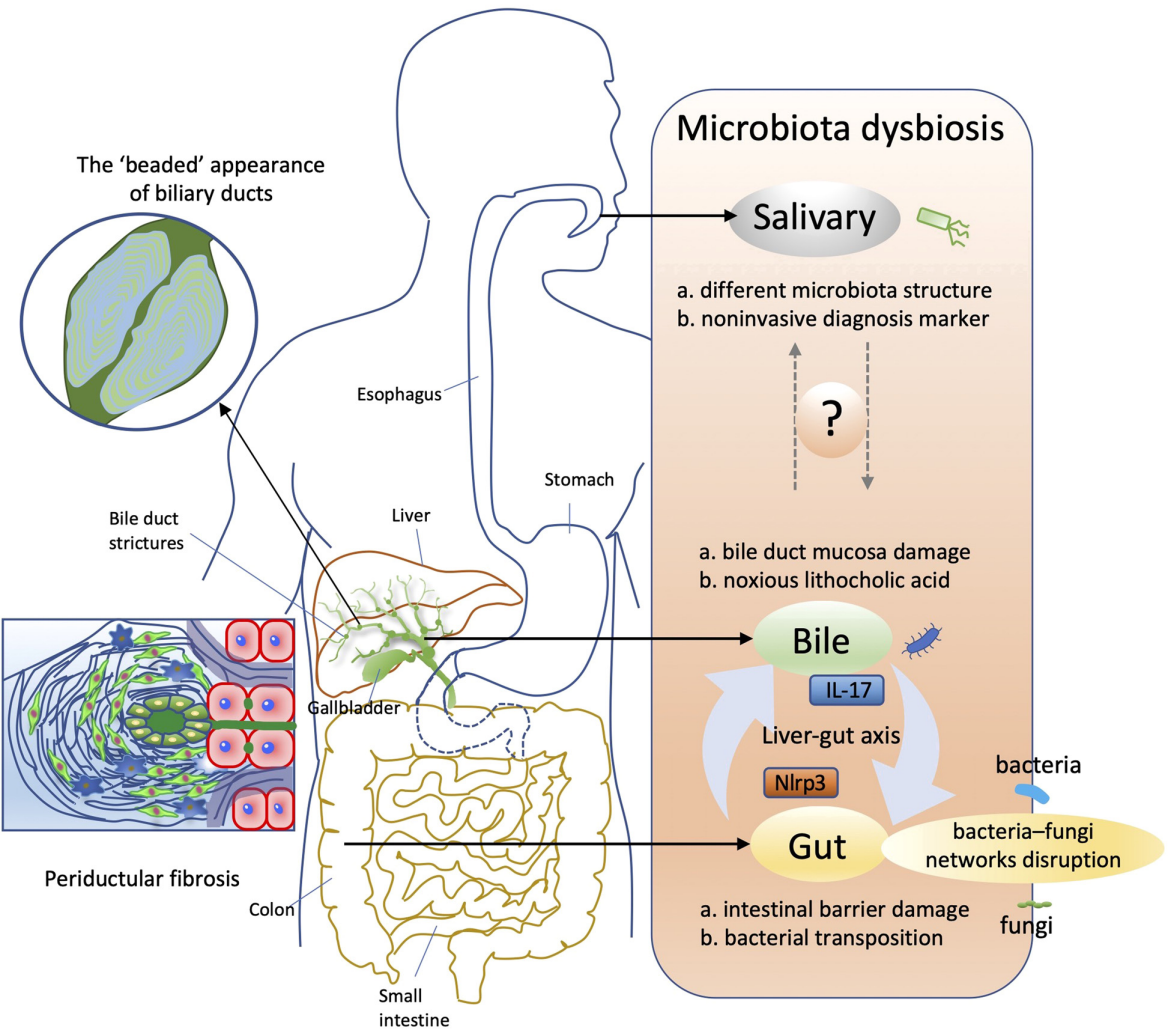
PSC and microbiota dysbiosis. PSC shows a characteristic “beaded” appearance of biliary ducts as a pattern of alternating strictures and dilatations. It pathologically characterises as periductular fibrosis. Dysbiosis of microbiota in the gut, bile, and saliva contribute to the pathogenesis of PSC. The liver–gut axis bridges the path for microbiota in bile and gut. On one hand, bacterial species disrupt the intestinal epithelial barrier and bacterial products activate the Nlrp3 inflammasome via the liver–gut axis, inducing Th17 priming in the liver. On the other hand, the bile microbiome contributes to PSC by increasing damage to the bile duct mucosa, increasing the concentration of noxious lithocholic acid in bile fluid, and changing the bile acid composition and flow which damages the intestines and impairs the intestinal barrier integrity, causing a vicious cycle that results in gut microbiota dysbiosis. Strong disruptions in bacteria–fungi networks found in gut faecal suggest a function of fungi in PSC. Salivary microbiota may contribute to biomarkers as a non-invasive diagnostic tool for PSC, but its pathogenic mechanism is still unclear.

Primary sclerosing cholangitis (PSC) is a cholangiopathy that affects both intrahepatic and extrahepatic biliary ducts. It is associated with chronic, progressive biliary inflammation, and fibrosis [1,2]; affected patients are at high risk of developing cholangiocarcinoma [3,4]. Although incidence and prevalence rates for PSC are infrequently reported, both rates appear to be increasing [5]. The causes of PSC are classified as genetic, with more than 20 susceptibility loci for PSC reaching genome-wide significance [6], and as microenvironmental, including diet, microbial imbalance, infection, and cholestasis [2]. Regardless of cause, the pathophysiological changes in PSC share a common phenotype of cholangiopathies, characterised by varying degrees of cholestasis, biliary hyperplasia, ductopenia, inflammation, and fibrosis in portal and periportal areas [7], which can progress to cirrhosis and end-stage liver disease. PSC is characterised by a “beaded” appearance of the biliary ducts (Figure 1), caused by periductal fibrosis [2], with a pattern of alternating strictures and dilatations in the large bile duct that is detectable via cholangiography [8]. Histologically, PSC is characterised by fibro-inflammatory destruction of the interlobular bile ducts, often with concentric layers (“onion skin”) of fibrosis (Figure 1) [6,8]. Liver transplantation is currently the best therapeutic option in cases of end-stage liver disease, because curative options for this disease are limited and often ineffective [6,8,9] and the use of ursodeoxycholic acid in the treatment of PSC is still controversial [10].

There is growing evidence that dysbiosis of microbiota contributes to the pathogenesis of PSC supported by the following findings: (a) concomitant ulcerative colitis (UC) and inflammatory bowel disease (IBD) are commonly linked to PSC [11]; (b) recurrence of PSC can be prevented by colectomy following liver transplantation [12]; (c) portal bacteraemia [13] and elevated levels of endotoxin in biliary cells are frequently observed in affected patients [14]; and (d) according to genome-wide association studies, the overall genetic architecture of PSC shares features with IBD and autoimmune diseases [6]. These data support the idea that liver–gut communication, the so-called enterohepatic circulation or gut–liver axis, could help to elucidate the pathogenesis of PSC [15]. Moreover, next generation sequencing studies have revealed faecal and mucosal alterations in the gut bacterial microbiome in patients with PSC that differ from both healthy individuals and patients with UC [16–21]. In addition to the gut [22], several recent studies have shown that patients with PSC harbour impaired microbiota in bile [23] and saliva [21,23], characterised by decreased biodiversity and an altered composition. A possible role of fungi in the pathogenesis or progression of PSC has also been reported [24]. Although the association between PSC and microbiota is strong, the underlying mechanisms of the disease are still unclear. Thus, the first goal of this review is to summarise this possible association in consideration of novel therapies.

Since both genetic and environmental factors contribute to the development and progression of PSC, the molecular machinery of cholangiocytes probably contributes to this gene–environment association [25]. Cholangiocytes undergo alterations during the progression of cholangiopathies, which may initiate and perpetuate inflammatory cascades; consequently, immune cells and fibroblasts in the periductal stroma are activated, which eventually leads to the characteristic fibro-inflammatory phenotype of this disease [25]. Under normal physiological conditions, bile is modified through the transport of water, ions, and solutes [26]. Under injurious conditions or a harsh environment, cholangiocytes can be activated [27], increasing cell proliferation and the secretion of pro-fibrotic and pro-inflammatory mediators [25]. In this setting, activated cholangiocytes promote hepatobiliary repair and the recruitment of various innate professional antigen-presenting cells [25]. Some persistently injured cholangiocytes enter a state of cellular senescence, characterised by cessation of the cell cycle and a transition to a hypersecretory, pro-inflammatory state referred to as senescence-associated secretory phenotypes (SASPs) [25,28,29]. Such an inflammatory fibrotic environment, when persistent, leads to sclerosing cholangitis [25]. Additionally, cholangiocytes can acquire a neuroendocrine-like phenotype in response to injury. The main neuroendocrine factors involved in the cholangiocyte response to injury include secretin, vascular endothelial growth factor, follicle-stimulating hormone, histamine, oestrogens, nerve growth factor, serotonin, and melatonin [30]. Furthermore, recent research has revealed new factors expressed by cholangiocytes in PSC, which may inform the development of new treatments of cholangiopathies. The second goal of this review is to summarise these new factors expressed by cholangiocytes during PSC progression.

Reliable, well-defined, and easily reproducible animal models help to provide insights into the pathophysiological processes and pathogenesis of liver disease [31,32]. Several models have been established to study the pathogenesis of PSC and identify potential molecular targets for therapy, including widely utilised chronic *in vivo* mouse models such as the model of multidrug resistance 2 deficiency (*Mdr2* ^−/−^) [33], the model of 3,5-diethoxycarbonyl-1,4-dihydrocollidine (DDC) [34], the recently reported *in vivo* model of acute PSC (intrabiliary instillation of second mitochondrial activator of caspases [SMAC] mimetic) [35], and *in vitro* models, such as those for organoids [36] and cholangioids [37]. Because of the complex, multifactorial nature of PSC, no other animal models developed so far have encompassed all the characteristic features of this disease [38]. Thus, the development of new animal models for PSC is crucial for acquiring pathogenic insights and testing therapeutic strategies. The third goal of this review is to provide an overview of the current and most available models for studying PSC and biliary fibrosis, including *in vivo* animal models of both acute and chronic disease and *in vitro* models of organoids and cholangioids.

## 2 Dysbiosis of microbiota in the pathogenesis of PSC

Dysbiosis of microbiota, especially gut microbiota, contributes to the pathogenesis of PSC. Recent reports have revealed novel information regarding the function of gut microbiota, alterations in both the bile microbiome and salivary microbiota in PSC, and the possible role of fungi in the pathogenesis or progression of PSC (Figure 1). In this section, we describe recent reports about microbiota dysbiosis in PSC (Table 1).

**Table 1:** Recent reports on mechanisms of microbiota in PSC

Dysbiosis	Mechanisms/impacts	Reference
Gut microbiota	Multiple bacterial species collaboratively disrupt the intestinal epithelial barrier and induce Th17 priming in the liver	[22]
Gut microbiota	Cholestasis is critical in inducing gut dysbiosis and enrichment of *Lactobacillus*. Translocation of *Lactobacillus* to the liver due to increased intestinal permeability induced activation and expansion of γδ T cells producing IL-17	[39]
Gut microbiota	Promotes intestinal barrier dysfunction and increases bacterial translocation amplifying the hepatic Nlrp3-mediated innate immune response	[40]
Bile microbiota	Damages bile duct mucosa via potentially elevated concentration of noxious bile acid and lithocholic acid	[23]
Fungal microbiota	Increases proportion of *Exophiala* and decreases proportion of *Saccharomyces cerevisiae* leading to the loss of anti-inflammatory properties	[24]
Salivary microbiota	Decreases the abundance of *Rothia* and *Haemophilus,* while related mechanism is pending	[41]

Th17, T helper 17; IL-17, interleukin 17.

### 2.1 Gut microbiota

Gut microbiota are normally confined to the intestinal lumen. Leakage of gut microbiota through the epithelial barrier can elicit immune responses that resemble liver inflammation in PSC [42]. Leakage of microbiota following intestinal epithelial barrier disruption was thus believed to be the fundamental cause of cholangiopathies [42], but details were still unknown. More recent research implicated hepatic interleukin (IL)-17 [22,39] and nucleotide-binding oligomerisation domain-like receptors (NLR), such as NLR family pyrin domain-containing protein 3 (Nlrp3) [40], as mediators of the immune response to gut microbiota dysbiosis.

Nakamoto and colleagues [22] identified *Klebsiella pneumonia* in gut microbiota from patients with PSC and demonstrated that *K. pneumoniae* disrupts the epithelial barrier, which results in bacterial leakage and liver inflammatory responses. After faecal samples from PSC patients were implanted into gnotobiotic mice, specific bacteria responsible for the pathological leakage and subsequent T helper 17 (Th17) priming were identified in the mouse livers. Additionally, using a special organoid model, Nakamoto revealed that an intestinal epithelial barrier disrupted by multiple bacterial species collaboratively induced Th17 priming in the liver [22], thus demonstrating that IL-17 secretion caused by gut microbiota dysbiosis plays a key role in the mediation of PSC. Moreover, in *Mdr2* ^−/−^ mice, an alteration in the microbiota that contributes to IL-17 production is mediated by intrahepatic γδ T cells isolated from livers affected by PSC [39], but not from livers infected by hepatitis C virus. These results demonstrate that intrahepatic IL-17^+^ γδ T cells activated by gut microbiota dysbiosis have a pathogenic role in modulating liver injury in PSC. Notably, the Th17 immune response caused by PSC-derived microbiota was ameliorated via antibiotic treatment, which indicates that antibiotic treatment may be a therapeutic strategy for PSC [22].

In addition to IL-17, Nlrp3 is closely related to the augmented progress of PSC [40]. The Nlrp3 inflammasome, a multiprotein complex acting as an essential sensor at the host–microbe interface, orchestrates innate immune responses to infection and cell damage [43,44]. The functional roles of gut microbiota and liver–gut communication in the pathogenesis of PSC were elucidated through the use of the *Mdr2* ^−/−^ model, in which gut microbiota were identified as drivers of PSC. This finding indicates that the Nlrp3 inflammasome is an essential mediator of inflammation within the liver–gut axis [40]. In the *Mdr2* ^−/−^ mice model, disruption of the epithelial barrier was followed by pronounced macrophage infiltration, with high levels of Nlrp3 expression [40]. Bacterial products can activate the Nlrp3 inflammasome by providing first and second signals [43], resulting in cleavage of pro-IL-1β into its active form, which has pro-inflammatory functions [44]. A striking finding was that IDN-7314, a pan-caspase inhibitor, mitigated liver injury and recovered both the serum bile acid profiles and the cholestasis-associated microbiota signature by dampening Nlrp3 inflammasome activation. Thus, targeting Nlrp3 may represent a therapeutic option for PSC [40].

### 2.2 Bile microbiota

In addition to gut microbiota, alterations in the bile microbiome in PSC have also been reported [23]. Bile acids play a crucial role in the pathogenesis of PSC [2], serving as intermediators between host metabolism and inflammation of both the liver and gut within the enterohepatic circulation [45]. Bile acids also influence the composition of gut microbiota through their intrinsic antimicrobial properties [46]. Conversely, changes in microbiota composition in the gut affect the metabolism of microbial bile acids and thus influence biological activities mediated by the bile acid [45].

The bacteria ecological properties of ductal bile fluid between PSC patients and controls were recently investigated [23]. Liwinski and colleagues studied comprehensive bile acid profiles from PSC [23]. They found that the most significant alterations of dysbiosis in microbial communities were in the bile fluid of patients with PSC [23]. They hypothesised that changes in the biliary microbiome increased the damage to the bile duct mucosa, potentially contributing to the pathogenesis of PSC. The effects of noxious lithocholic acid in bile fluid may also contribute to this damage [23]. Alterations in the bile acid composition and flow significantly damaged and impaired the integrity of the intestinal barrier [40], causing a vicious cycle that resulted in gut microbiota dysbiosis. Therefore, ductal microbial dysbiosis in biliary fluid may further pathophysiological progression of PSC. The precise regulation of biliary microbial colonisation could help to reduce the risk of adverse outcomes linked to PSC.

### 2.3 Fungal microbiota

A possible role of fungi has been indicated by numerous observations in the pathogenesis or progression of PSC. First, PSC patients often have high levels of anti-*Saccharomyces cerevisiae* antibodies [47]. Second, bile from PSC patients can be colonised by *Candida albicans*, and has been associated with a poor prognosis [48]. Third, patients with genetic variants of caspase recruitment domain-9, a protein associated with innate immunity against fungi, had increased susceptibility to both PSC and IBD [49]. In line with these findings, Lemoinne and colleagues [24] compared the fungal microbiota in patients with PSC, some of whom also had IBD, with that of patients with IBD alone and healthy controls. Their data showed, for the first time, that PSC patients displayed a relative increase in biodiversity and altered composition of fungi with gut dysbiosis. They also found an elevated proportion of *Exophiala* species, which likely participates in the pathophysiology of PSC [50], and a lowered proportion of *S. cerevisiae,* therefore a loss of anti-inflammatory properties [51]. Compared with the gut microbiota of patients with IBD and healthy controls, those of patients with PSC showed a strong disorder in fungi–bacteria networks. The contribution of gut fungi to the pathogenesis of PSC should therefore be considered a promising therapeutic target. However, the causes for disruption of the bacteria–fungi networks are far from elucidated, and the mechanism of fungal dysbiosis in PSC is still unknown.

## 3 Altered cholangiocyte responses in PSC

**Figure 2:**
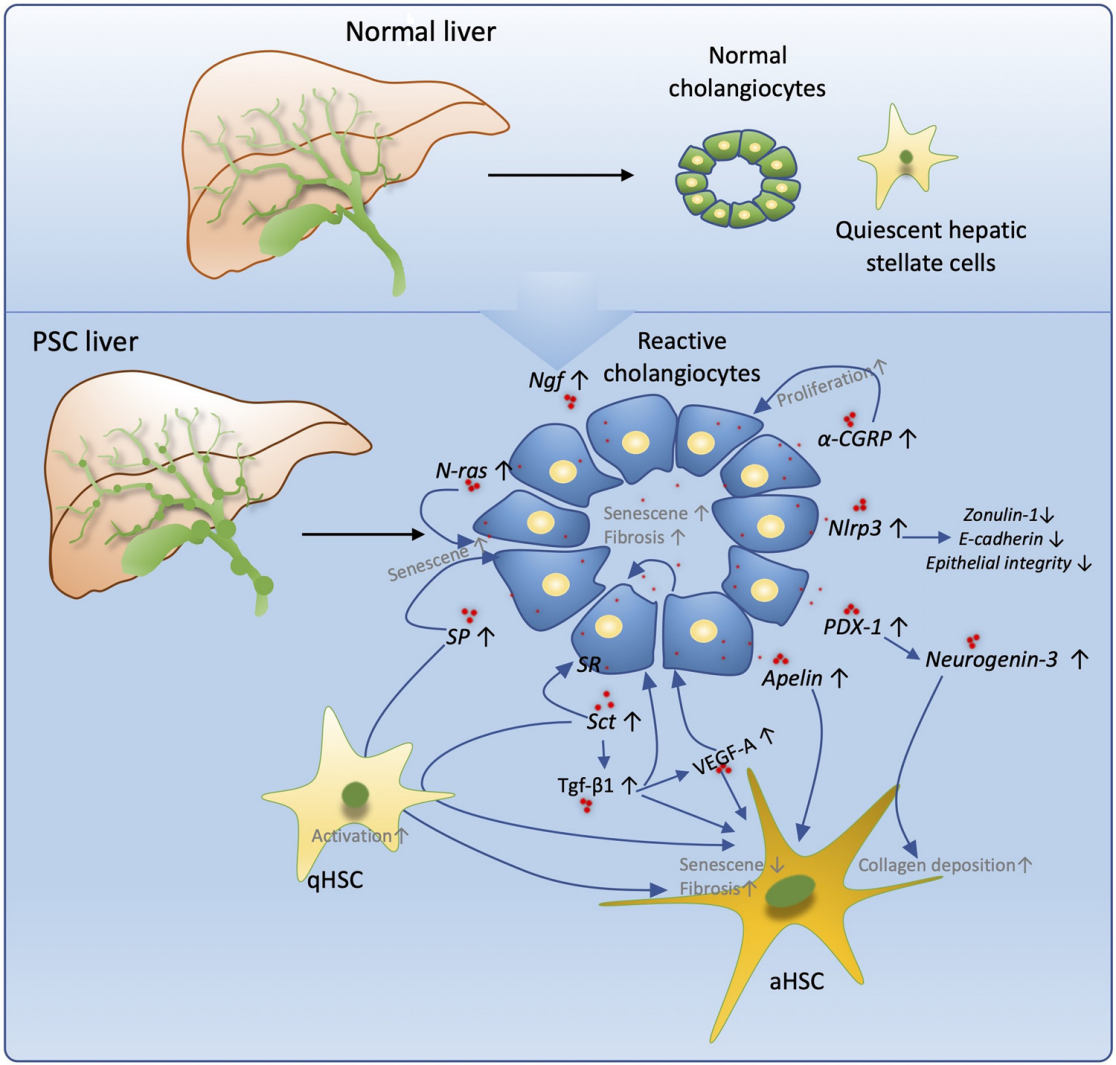
Key factors expressed by reactive cholangiocytes in PSC. In the normal liver, cholangiocytes and HSC stay in normal or quiescent states. In the PSC liver, the cholangiocytes undergo activation and secrete factors, such as secretin, TGF-β1, VEGF-A, apelin, PDX-1, Nlrp3, α-CGRP, Ngf, N-Ras, and SP. These factors contribute to senescence or proliferation of cholangiocytes or result in damage to biliary integrity via an autocrine manner. These reactive factors may cause activation of quiescent HSC and decrease HSC senescence but increase fibrosis via collagen deposition. α-CGRP, α-Calcitonin gene-related peptide; Tgf-β1, Transforming growth factor-β1; Sct, secretin; SR, secretin receptor; VEGF-A, vascular endothelial growth factor-A; HSCs, hepatic stellate cells; Nlrp3, pyrin domain-containing protein 3; PDX-1, pancreatic duodenal homeobox protein 1; N-Ras, neuroblastoma Ras. Ngf: nerve growth factor; SP: Substance P. qHSC, quiescent HSC; aHSC, active HSC.

Cholangiocytes, key target cells in cholangiopathies [15,52,53], are known to have phenotypic alterations in PSC. They acquire a neuroendocrine-like phenotype in response to injury, which includes upregulation of secretin [54], vascular endothelial growth factor [55], follicle-stimulating hormone [56], histamine [57], oestrogens [58], nerve growth factor [59], serotonin [60], and melatonin [61]. Synthesised by reactive cholangiocytes, these factors are reported to modulate biliary damage by autocrine or paracrine mechanisms, or both [30]. Additionally, recent research has revealed new factors expressed in cholangiocytes in PSC (Table 2), which may accelerate the development of new therapeutic options for cholangiopathies (Figure 2).

**Table 2:** Summarising the expressed factors involved in cholangiocyte in PSC

Factor/mediator	Functions	Reference
Secretin	Upregulate TGF-β1 expression in cholangiocyte via SR, directly stimulate biliary cells senescence, and activate HSCs, and promote hepatic fibrosis via TGF-β1 biliary secretion	[62]
	Increase TGF-β1 biliary secretion (via secretin/SR/microRNA 125b axis), VEGF-A expression, which subsequently increases cholangiocyte senescence (in autocrine manner), and fibrogenic activity, and decrease HSCs senescence (in paracrine manner)	[63]
	Promote cholangiocyte proliferation in cholestasis by reducing microRNA let-7a expression, resulting in upregulation of nerve growth factor	[64]
Nlrp3	Stimulate IL-18 expression, decrease Zonulin-1 and E-cadherin expression, synthesis of proinflammatory cytokines, and influence epithelial integrity of cholangiocytes	[65]
PDX-1	Expressed by reactive cholangiocyte, act as a major determinant of cholangiocyte proliferation in response to cholestatic injury, regulated by Hes-1 downregulation	[66]
	Activates neurogenin-3 expression resulting in cholangiocyte proliferation	[67]
Neurogenin-3	Expressed in proliferating cholangiocytes, ﻿regulates cholangiocyte proliferation via activation of microRNA-7a and regulates IGF-1 synthesis and ﻿collagen deposition	[67]
Substance P	Promotes biliary senescence, peribiliary inflammation, and hepatic fibrosis by increased microRNA-31, stimulates the release of SASPs and TGF-β1, leading to activate HSCs by increasing HSCs fibrosis and reduced HSCs senescence	[68,69]
Apelin	Induces cholangiocyte proliferation via Nox4/ROS/ERK signalling pathway, and induces HSC proliferation and activation via ROS	[70]
α-CGRP	Stimulates cholangiocyte proliferation, reduces cellular senescence of HSCs, increases activation of p38, and JNK/MAPK signalling pathway	[71]
N-Ras	Induces senescence of cholangiocytes; expresses SASPs components as proinflammatory cytokines (e.g., IL-6), chemokines (e.g., IL-8) and profibrotic mediators (e.g., PAI-1)	[72]

α-CGRP, α-Calcitonin gene-related peptide; TGF-β1, Transforming growth factor-β1; SR, secretin receptor; VEGF-A, vascular endothelial growth factor-A; HSCs, hepatic stellate cells; SASPs, senescence-associated secretory phenotypes; ROS, reactive oxygen species; Nlrp3, pyrin domain-containing protein 3; PDX-1, pancreatic duodenal homeobox protein 1; IGF-1, insulin growth factor-1; IL, interleukin; SASPs: senescence-associated secretory phenotype; Nox4, NADPH Oxidase 4; ERK, extracellular signal-regulated kinase; JNK, c-Jun N-terminal kinases; MAPK, mitogen-associated protein kinase; PAI-1, plasminogen activator inhibitor-1; N-Ras, neuroblastoma Ras.

### 3.1 Secretin

Expressed by large cholangiocytes, secretin exerts its effects through secretin receptors (SRs) [62,64,73,74]. In cholestatic models, enhanced cholangiocyte proliferation is associated with elevated expression of SR in cholangiocytes and enhanced cyclic adenosine 3′,5′-monophosphate-dependent secretin-stimulated ductal secretion [61]. By contrast, knockout SR (SR^−/−^) [75] or knockdown secretin (secretin^−/−^) [54] reduces cholangiocyte hyperplasia which indicates that secretin and SR are essential trophic regulators that sustain biliary growth. Similarly, secretin and SR were more strongly expressed in PSC liver samples [64]. In the *Mdr2* ^−/−^ model of PSC, secretin produced by cholangiocytes decreases microRNA let-7a expression [64], which results in the upregulation of nerve growth factor and thereby enables the proliferation of cholangiocytes [59]. This activation of biliary proliferation effected by secretin is believed to initiate and progress hepatic fibrosis via a paracrine manner by activating hepatic stellate cells (HSCs) and autocrine profibrogenic factors.

A recent study showed firm evidence of an autocrine role of secretin in the modulation of senescence of biliary cells. A cholestasis-induced ductular reaction stimulates the secretion of secretin and, through SR, causes increasingly high levels of transforming growth factor-β1 (TGF-β1) in cholangiocytes [62]. Because of the high secretion of TGF-β1 by cholangiocytes, the TGF-β1 receptor promotes senescence in cholangiocytes but increases the activation of a profibrogenic phenotype in HSCs [62]. Secretin-induced biliary secretion of TGF-β1 results in cholangiocyte senescence through an autocrine loop, which in turn increases the release of SASPs, including TGF-β1, and triggers hepatic fibrosis by decreasing HSC senescence [62]. Moreover, a limited ductular reaction was shown by knockdown of the secretin/SR axis and was followed by compensatory cholangiocyte senescence and reduced levels of TGF-β1; thus, the secretin/SR axis may be regarded as a potential target for this disease.

Another study also demonstrated a key function of SR in the modulation of bile duct damage and hepatic fibrosis [63]. Researchers found that by knockdown of SR in PSC mice, the normally increased ductular reaction, hepatic fibrosis, and angiogenesis were reduced in SR^−/−^ PSC mice, thereby demonstrating a therapeutic effect by targeting SR. Moreover, levels of senescence among cholangiocytes in PSC mice decreased to normal by the SR knockdown [63], which in turn reduced bile duct damage and hepatic fibrosis in PSC mice. Additionally, secretin-induced TGF-β1 secretion was found to be mediated by microRNA-125b [63]. The knockout of the secretin/SR axis in PSC mice was associated with increased biliary expression of secretin-dependent microRNA-125b [64] and subsequent reduced expression of microRNA-125b-dependent vascular endothelial growth factor-A, whereas vascular endothelial growth factor-A was believed to increase cholangiocyte senescence and fibrosis and to decrease the HSC senescence but increase the fibrogenic activity [55].

### 3.2 Nlrp3

As described previously, bacterial products can function as a signal to activate the Nlrp3 inflammasome; thus, the Nlrp3 inflammasome is an essential mediator of inflammation within the gut–liver axis [40]. On activation, the inflammasome complex cleaves pro-IL-1β into its active form, which exerts pro-inflammatory functions [44]. One study has demonstrated the expression of the Nlrp3 inflammasome in proliferating cholangiocytes, which led to the synthesis of pro-inflammatory cytokines in cholangiocytes [65]. In a model of PSC, Nlrp3 activation affected the biological response of cholangiocytes to injury, and the development and progression of liver injury [65]. Nlrp3 and its inflammasome components, such as apoptosis-associated speck-like protein, were upregulated in reactive cholangiocytes isolated from a PSC model induced by DDC [65]. Cultured cholangiocytes stimulated by lipopolysaccharides and adenosine triphosphate increased the expression of Nlrp3 and pro-inflammatory cytokines IL-18 and IL-6, but not IL-1β, in cholangiocytes [65]. Additionally, incubation with lipopolysaccharides and adenosine triphosphate was able to significantly decrease E-cadherin and zonulin-1 expression in cultured cholangiocytes, which indicates the influence of Nlrp3 on the epithelial integrity of cholangiocytes [65]. In a model of knockdown Nlrp3 in cultured cholangiocytes, these effects of lipopolysaccharides and adenosine triphosphate were completely abolished. This provides further evidence of the effects of Nlrp3 in cholangiocytes on the cell adhesion molecules involved in epithelial cell barrier functions. In other words, these results showed that the activation of the Nlrp3 inflammasome mediated by bacterial products known to have pathogen-associated molecular patterns, such as lipopolysaccharides, influences the epithelial barrier function of biliary cells *in vitro.* This finding supports the concept that microbial products could alter the biological function of cholangiocyte.

### 3.3 PDX-1

Pancreatic duodenal homeobox protein 1 (PDX-1) is a transcription factor for physiological and pathophysiological processes in the pancreas [76]. In PSC, PDX-1 may also be expressed by reactive cholangiocytes [66]. In isolated cholangiocytes from PSC patients, PDX-1 is overexpressed, whereas the protein hairy and enhancer of split 1 (Hes-1), an effector that is important for the development of the biliary tree, is downregulated [66]. This shows that PDX-1/Hes-1 interactions determine the proliferation of biliary cells in response to injury, which may have implications for the treatment of sclerosing cholangitis [66]. Additionally, PDX-1-dependent activation of neurogenin-3 initiates cholangiocyte proliferation [67]. Furthermore, microRNA-7a is hyperexpressed in proliferating cholangiocytes and mediates neurogenin-3 effects, which demonstrates that cholangiocyte proliferation is regulated by the neurogenin-3-dependent activation of microRNA-7a [67]. Neurogenin-3, as an effector of PDX-1, which is regulated by Hes-1, may thus play a significant pathophysiological role in chronic PSC by activating microRNA-7a.

### 3.4 Substance P

Serum levels of substance P are elevated in patients with PSC [68]. By interacting with neurokinin-1 receptor (NK1R), substance P is involved in the proliferation of cholangiocytes in mice in which the bile ducts are ligated [77] and in cholangiocarcinoma growth in humans [78]. Wan and colleagues showed that excessive substance P increases hepatic fibrosis by differential alterations in the senescence of cholangiocytes and HSCs [68]. More recently, in a PSC model, Ceci and colleagues [69] demonstrated that cholangiocytes secrete substance P, which promotes senescence of biliary cells, peribiliary inflammation, and liver fibrosis. Substance P in turn stimulated the release of SASPs and TGF-β1, thus activating HSCs and reducing HSC senescence [69]. Additionally, the expression of microRNA-31 was elevated in PSC mice and reduced in NK1R^−/−^ PSC mice; this finding indicates that substance P/NK1R or microRNA-31, or both may be potential targets in treating human PSC [69].

### 3.5 Apelin

Recently published research provides evidence that apelin and its G-protein-coupled receptor, apelin receptor, triggers cholangiocyte proliferation and liver fibrosis in mouse models of cholestasis [70]. Apelin serum levels and biliary expression of apelin and its receptor also increased in PSC samples [70]. Serum levels of apelin and hepatic expression of apelin and its receptor were reported to increase in cirrhotic rats [79]. Apelin binding to its receptor triggered biliary damage via the Nox4/reactive oxygen species/extracellular signal-regulated kinase signalling pathway, and induced HSC activation through changes in the reactive oxygen species levels [70]. Therefore, the modulation of the apelin/apelin receptor axis may represent a novel target for fibrosis in PSC.

### 3.6 α-CGRP

In parallel to the role of substance P in biliary functions, α-calcitonin gene-related peptide (α-CGRP), a 37-amino acid neuropeptide, plays an essential role in cholestatic liver injury by differentially regulating cellular senescence of HSCs and cholangiocytes [71]. In one study, α-CGRP levels were higher in the serum of cirrhotic patients than in that of healthy controls [80]. In another study, α-CGRP serum levels and liver mRNA expression of calcitonin related polypeptide alpha (*Cacla)* (encoding α-CGRP) and α-CGRP receptor components were higher in late-stage PSC samples than in healthy control samples [71]. Depletion of α-CGRP reduced liver injury and fibrosis in association with enhanced cellular senescence of HSCs, reduced senescence of cholangiocytes, and decreased activation of p38 and c-Jun N-terminal protein kinase in mitogen-activated protein kinase signalling pathways [71]. Together, these data demonstrated that endogenous α-CGRP facilitated cholestatic hepatic fibrosis through differential alterations in senescence of HSCs and cholangiocytes via the activation of p38 and c-Jun N-terminal protein kinase signalling. Moreover, abolishing α-CGRP decreased senescence in an intact liver and isolated cholangiocytes, but increased senescence in HSCs from mice in which the bile ducts were ligated [71]. Therefore, the modulation of α-CGRP/α-CGRP receptor signalling may also be key to managing biliary senescence and liver fibrosis in PSC.

### 3.7 N-Ras

N-Ras is known to be an inducer of senescence. A previous study showed that N-Ras-related biliary senescence is a characteristic of PSC [72]. N-Ras levels increased in PSC cholangiocytes and in cultured cholangiocytes following experimentally induced senescence [72]. Senescent biliary cells can then become SASPs, a potentially pathologic state characterised by hypersecretion of pro-inflammatory cytokines, chemokines, and pro-fibrotic mediators (such as IL-6, IL-8, and plasminogen activator inhibitor-1) [72]. The suppression of N-Ras abrogated experimentally induced SASPs and biliary senescence [72]. These secreted mediators altered the SASP cell microenvironment, reinforced the senescent phenotype, and exacerbated injurious fibro-inflammatory responses that, in the liver, results in progressive damage and ultimately chronic PSC.

The cholangiocyte reactions of hyperplasia, proliferation, and senescence are also promoted by several functional factors interacting with their specific receptors, such as nerve growth factor, follicle-stimulating hormone, gonadotropin-releasing hormone, oestrogens, and biogenic amine histamine [30]. These studies provide evidence that regulations of different factors and their receptors could be of prime importance in management of the balance between biliary growth and loss in biliary diseases, especially in PSC. As described previously, factors secreted by proliferative or senescent cholangiocytes may play a role in the pathogenesis of PSC. These factors and their receptors, in turn, are potential therapeutic targets in PSC.

## 4 Commonly used models for studying PSC

**Figure 3:**
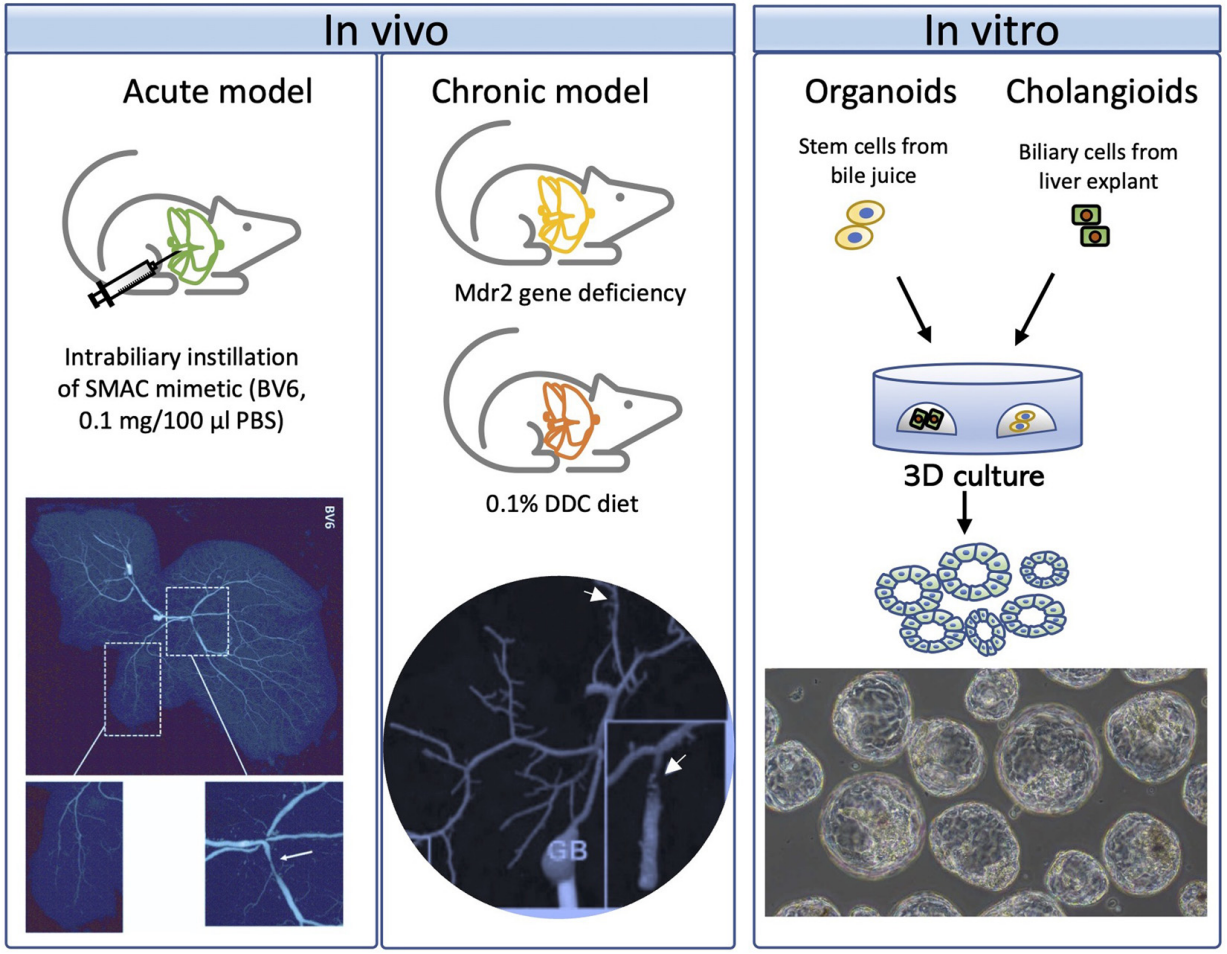
Commonly used PSC models. Established models include the chronic *in vivo* models such as the Mdr2^−/−^ model and the DDC model, the recently reported *in vivo* model of acute PSC via intrabiliary instillation of a SMAC mimetic, and *in vitro* models such as organoids and cholangioids. A copy of cholangiography images for the acute model was acquired from Guicciardi et al. [35]. The arrow in the right panel shows a stricture in an intrahepatic duct and adjacent dilatation. The left panel shows damage and loss of small bile ducts. A copy of cholangiography images for the DDC model was acquired from Fickert et al. [34]. Slight dilatation of bile ducts (arrows) is present in the DDC model. The *in vitro* model cells should be obtained from the bile or liver of PSC and 3D culture system are needed. The phase-image shows the morphologies of the organoids or cholangioids. GB, gall bladder.

Reliable, well-defined, and easily reproducible animal models provide insights into the pathophysiological processes and pathogenesis of liver disease [31,32]. The development of new models of PSC, therefore, is still crucial. No animal model of PSC was available a decade ago﻿, as pointed out by Pollheimer [38]; since then, several models have been established, including widely utilised chronic *in vivo* models such as the Mdr2^−/−^ [33] and DDC models [34], the recently reported *in vivo* model of acute PSC [35], and *in vitro* models such as organoids [36] and cholangioids [37]. In this section, we provide an overview of the available models for studying PSC and biliary fibrosis, including both *in vivo* and *in vitro* animal models (Table 3 and Figure 3).

**Table 3:** Commonly used models for studying the PSC

Type	Methods	Characteristics	Reference
*In vivo* model: acute PSC	Intrabiliary instillation of SMAC mimetic (BV6, 0.1 mg/100 μL PBS)	TRAIL-dependent acute sclerosing cholangitis	[35]
		Requires an abdominal surgery	
*In vivo* model: chronic PSC	Mdr2 gene deficiency	Disrupts tight junctions and basement membranes, causing bile acid leakage into portal tracts with consecutive periductal inflammation and liver fibrosis leading to biliary cell death	[33]
*In vivo* model: chronic PSC	0.1% DDC diet	Formation of intraductal porphyrin plugs	[34]
		Can recover with DDC withdrawal	
		Handle simply with oral intake	
*In vitro* model: organoids	Stem cells from the bile juice of PSC via ERCP	Expresses a biliary genetic phenotype with known cholangiocyte markers	[36]
	3D culture with Matrigel	Retain the ability to react to inflammatory stimuli by secreting chemokines and propagating an immune-reactive phenotype reflective of the pathogenesis of PSC	
*In vitro* model: cholangioids	Biliary cells from liver explants of PSC	Retains cholangiocyte phenotype and are functionally active	[37]
	3D culture with Matrigel	Exhibits cellular senescence and increases the SASPs expression (IL6, p21, p16, SA-β-gal, and yH2A.x)	

TRAIL, TNF-related apoptosis-inducing ligand; SASPs, senescence-associated secretory phenotypes; SA-β-gal, senescence-associated β-galactosidase; SMAC, second mitochondrial activator of caspases; DDC, 3,5-diethoxycarbonyl-1,4-dihydrocollidine; IL, interleukin.

### 4.1 Acute animal model

#### 4.1.1 Intrabiliary instillation of SMAC mimetic

Acute cholangitis may be induced by SMAC mimetics, also referred to as BV6, through a single-dose instillation into the biliary system in mice [35]. Treated mice displayed apoptosis of cholangiocytes, identified at 12 h after instillation [35]. After this acute reaction, the mice displayed progressive cholestatic damage resembling PSC in humans, presenting as classic concentric “onion skin” bile ducts within portal tract areas, which peaked at day 5 after surgery [35]. These manifestations may be completely reversed within 3 weeks [35], which is indicative of an auto-recovery effect. The authors pointed out that the activation of tumour necrosis factor (TNF)-related apoptosis-inducing ligand (TRAIL) and the TRAIL receptor signalling pathway is required for this model. In this setting, the depletion of the TRAIL gene may protect against SMAC mimetic-induced cholangiopathy, as evidenced by TRAIL^−/−^ mice that were highly resistant to SMAC mimetic-induced hepatic injury and displayed normal histological features in the liver [35]. Therefore, this model is suitable for studying TRAIL, its receptor, and cellular inhibitors of apoptosis proteins (such as cIAP-1 and cIAP-2), which are reportedly negative regulators of inflammation and TRAIL receptor signalling [81,82]. However, the abdominal manipulation during intrabiliary instillation is difficult for inexperienced surgeons.

### 4.2 Chronic animal model

#### 4.2.1 Mdr2^−/−^ model

A series of studies have previously documented depletion of the Mdr2 gene ( *Abcb4*) in mice (Mdr2^−/−^) as a reproducible model of spontaneously progressive chronic fibrotic biliary disease, in which histological lesions closely resemble those of human PSC [83–86]. *Abcb4* functions in transporting phospholipids from liver hepatocytes into bile [87]. The knockdown of this gene eliminates phospholipid secretion into bile. The lack of biliary phospholipids could result in toxic bile acid-induced biliary injury and ultimately lead to sclerosing cholangitis [84]. Moreover, bile ducts in Mdr2^−/−^ mice showed disruptions of tight junctions and basement membranes, which resulted in bile acid leakage into portal tracts, induction of a portal CD11b- and CD4-positive inflammatory infiltrate, and activation of pro-inflammatory cytokines (such as TNF-α and IL-1β) and profibrogenic cytokines (such as TGF-β1) [33]. This in turn could activate periductal myofibroblasts and lead to periductal fibrosis, separation of the peribiliary plexus from biliary epithelial cells, and finally, atrophy and death of the biliary epithelium [33]. In other words, abolishing Mdr2 resulted in leakage of bile acids from the bile ducts into the portal tracts, which led to periductal inflammation followed by fibrosis. In the Mdr2^−/−^ model of PSC, the composition of ductal bile and biliary integrity may play key roles in PSC. Thus, this model is more suitable for studying factors that influence biliary composition and impair biliary integrity, leading to PSC, such as pathogens from microbiota that cause biliary damage [40]. Although Mdr2^−/−^ mice evince several key characteristics of human PSC (including the development of cholangitis, irregular biliary formation, and hepatic fibrosis), their condition does not mimic human disease, because humans with PSC do not have defects in this gene or loss of biliary phospholipid secretion into bile [35]; these are the main shortcomings of this model.

#### 4.2.2 DDC model

Approximately two decades ago, mice fed with DDC [88] were reported to represent a model of xenobiotic-induced cholangiopathy [34,89]. DDC feeding led to increased biliary porphyrin secretion, which resulted in the expression of cytokines and molecules, such as osteopontin, vascular cell adhesion molecule, and TNF-α, in cholangiocytes [34]. Chronic administration of DDC in mice reproduced the main histopathological hallmarks of human PSC found in extrahepatic biliary ducts with histological lesions. These hallmarks included: (a) the rebuilding of biliary compartments, which provoked reactive phenotypes of cholangiocytes [34]; (b) periductular and biliary fibrosis with inflammatory infiltrate [34,90]; and (c) proliferation of peribiliary glands and mucinous metaplasia [91]. This model is therefore useful for investigating mechanisms of chronic cholangiopathies and their sequelae, including liver fibrosis of the biliary type, ductular reaction, and peribiliary gland proliferation, and for testing novel therapeutic strategies for these diseases. Conversely, DDC-fed mice automatically recover upon DDC withdrawal; thus, this model can be used to observe recovery after discontinuing the toxic agent under controlled conditions [91].

### 4.3 *In vitro* model

#### 4.3.1 Organoids

In addition to animal models, *in vitro* models, such as cell organoids, have also been developed to better delineate the pathogenesis of PSC and identify new therapies for liver diseases [32,92–94]. One study showed that biliary cells from bile duct tissues could be used to generate biliary organoids [95]. Soraka and colleagues developed three-dimensional organoids using cells from the bile of patients with PSC who underwent endoscopic retrograde cholangiopancreatography [36]. These bile-derived organoids with a biliary phenotype could secrete cytokines [36]. Additionally, bile-derived organoids retain normal responses to biliary diseases, including the ability to react to inflammatory stimuli by secreting chemokines and to propagate an immune-reactive phenotype that reflects the pathogenesis of each disease [36]. Thus, bile-derived organoids enable the investigation of PSC pathogenesis and pharmacotherapeutic interventions using cells from affected patients.

#### 4.3.2 Cholangioids

Tabibian and colleagues isolated and cultured cholangiocytes derived from PSC patients and demonstrated that these cholangiocytes exhibited characteristics of cellular senescence and secreted SASP markers, including IL-6 and IL-8 [96]. Loarca and colleagues developed biliary spheroids, termed “cholangioids,” using cholangiocytes from patients with PSC and a gelatinous protein mixture (Matrigel) on dishes that were precoated with poly(2-hydroxyethyl methacrylate) to prevent cells from attaching to the dish surface [37]. The cholangioids retained the cholangiocyte phenotype and were functionally active. Additionally, after exposure to hydrogen peroxide, the cholangioids exhibited cellular senescence and SASPs with an increased expression of IL-6, p21, p16, senescence-associated β-galactosidase, and yH2A.x [37]. Thus, this *in vitro* cholangioid model mimicked several features of PSC and can be useful for studying the pathogenesis of PSC and identifying new therapeutic targets. However, cholangioids were established from cholangiocytes alone in the reported studies, and as Sato pointed out in a recent review [97], they are absent of supporting cells, such as endothelial cells, Kupffer cells, and HSCs, and therefore cannot imitate the *in vivo* environment and cell-to-cell interactions of cholangiocytes with other hepatic cells [97]. Nevertheless, this method, in combination with a co-culture system, may prove suitable to some extent in *in vitro* models and contribute to the testing of new drugs for PSC in the near future.

## 5 Conclusion and future perspectives

Research has revealed new information regarding PSC: (a) dysbiosis of microbiota, including bacterial and fungal microbiota in the gut, bile, and saliva, contributes to the pathogenesis of PSC, and related mechanisms of microbiota may contribute to the development of novel biomarkers and therapeutic targets of PSC pathogenesis; (b) cholangiocytes, whose reactions include hyperplasia, proliferation, or senescence, are promoted by several growth factors, and factors secreted by proliferative or senescent cholangiocytes may contribute to the pathogenesis of PSC, and along with their receptors, may be potential targets for the treatment of PSC; (c) because of the complex, multifactorial nature of PSC, no animal models developed so far recapitulate all the characteristic features of PSC, but several models could be helpful for studying the pathogenesis of PSC according to their purpose and even used in combination.

Synergistic insights into PSC arise from *in vitro* studies, animal models, genetic investigations, and evolving notions and experiments of the interactions between gut microbiota and immunological factors. Notably, in a recent research by Aloia et al. [98], biliary cells isolated from liver tissues of DDC-fed mice could be used to generate organoids within Matrigel [98]. Based on transcriptional profiles, Aloia and colleagues found that cells in organoids underwent genome-wide remodelling as a result of DDC-induced biliary damage, which may be crucial in transdifferentiation and liver regeneration [98]. A combination of organoid technology with mice fed with DDC thus provided a novel series of techniques for studying PSC. Treatment of PSC is currently confined to supportive measures, but advances in pathobiology suggest that new stratified approaches will soon be available.

## List of abbreviations

α-CGRP: α-calcitonin gene-related peptide
BDOs: bile-derived organoids
cIAP: cellular inhibitor of apoptosis protein
DDC: 3,5-diethoxycarbonyl-1,4-dihydrocollidine
HSCs: hepatic stellate cells
IBD: inflammatory bowel disease
IL: interleukin
Mdr2^−/−^
: multidrug resistance 2 deficiency
NK1R: neurokinin-1 receptor
Nlrp3: pyrin domain-containing protein 3
PSC: primary sclerosing cholangitis
PDX-1: pancreatic duodenal homeobox protein 1
SASPs: senescence-associated secretory phenotypes
SMAC: second mitochondrial activator of caspases
Sct: secretin
SR: secretin receptor
SR^−/−^
: knockout SR
SP: substance P
TGF-β1: transforming growth factor-β1
TNF: tumour necrosis factor
Th17: T helper 17
TRAIL: TNF-related apoptosis-inducing ligand
UC: ulcerative colitis;
